# How to Educate Learners to Manipulate Equipment in Trauma (HELMET): An Educational Workshop on Football Equipment Removal in Trauma Care

**DOI:** 10.7759/cureus.109747

**Published:** 2026-05-27

**Authors:** Jose E Gomez, Daniela Usuga, Bhaskar Thakur, Jason Vadhan, Jenna C Bryant, Dustin Harris

**Affiliations:** 1 Emergency Medicine, University of Texas Southwestern Medical Center, Dallas, USA; 2 Emergency Medicine/The Peter O'Donnell Jr. School of Public Health, Department of Health Data Science and Biostatistics, University of Texas Southwestern Medical Center, Dallas, USA; 3 Emergency Medicine, Methodist Dallas Medical Center, Dallas, USA

**Keywords:** emergency medicine training, football injury, spinal cord injury (sci), sports medicine curriculum, trauma and emergency

## Abstract

Introduction: American football carries a risk of traumatic injuries, including spinal cord injury (SCI). Proper management of suspected spinal injury in athletes, including safe equipment removal, is essential to prevent secondary injury. Emergency medicine (EM) physicians may encounter these scenarios, but often have limited formal training. This study evaluates EM residents’ prior training on this and assesses changes in self-reported confidence and comfort following a targeted educational intervention on football equipment removal.

Methods: We conducted a single-center, pre-post quasi-experimental study among EM residents at a three-year residency program. Participants completed a pre-intervention survey assessing prior exposure and confidence. They then underwent a brief educational intervention consisting of a video and a hands-on workshop on football equipment removal. A post-intervention survey measured changes in confidence and comfort using a 5-point Likert scale. Paired responses were analyzed using Wilcoxon signed-rank tests.

Results: Of 76 eligible residents, 29 participated and 23 completed both surveys (n=23/29, 79% completion rate). Most participants reported having minimal prior training or lecture on this topic (n=23/28, 82%). Following the intervention, there were statistically significant improvements in self-reported confidence across all domains, including timing of removal, cervical spine stabilization, airway access, and equipment removal (p < 0.001). Self-reported comfort in managing these trauma scenarios also improved (p < 0.001).

Conclusions: EM residents reported limited prior exposure to managing and treating suspected spine-injured athletes during residency training. A brief, simulation-based workshop significantly improved self-reported confidence and comfort in removing football equipment and may help address gaps in training for high-risk but infrequent clinical scenarios.

## Introduction

American football is widely played in the United States, with an estimated 5.6 million participants over the age of six and an incidence of approximately 16.17 per 1,000 high school athletes presenting to the emergency department (ED) for football-related injuries [1,2]. Due to the high-impact nature of the sport, there is an associated risk of traumatic injuries, including spinal cord injury (SCI). Sports-related SCI is the fourth most common cause, with adolescents, aged 14-17 years, having the highest incidence rates [3,4].

Although uncommon, appropriate management of athletes with suspected spinal injuries is critical to prevent secondary injury. Spine immobilization can be complicated by protective equipment such as helmets and shoulder pads, which are typically left in place until removal by trained providers in the ED [5]. This practice is supported by guidelines from organizations such as the National Athletic Trainers’ Association (NATA) [6,7].

While athletic trainers and team physicians are often proficient in football equipment removal, emergency physicians may be required to manage these patients when sports medicine personnel are unavailable or when emergent transport necessitates equipment removal in the emergency department. Although uncommon, these presentations involve core emergency medicine competencies, including trauma resuscitation, airway management, cervical spine stabilization, and procedural readiness. To date, little is known about emergency medicine residents' exposure to football equipment removal or their preparedness to manage athletes with suspected SCI, and there exists no EM-specific training or standardized curricula on this topic. The primary objective of this study was to evaluate changes in residents' self-reported confidence and comfort following a brief educational intervention. Secondary objectives included characterizing prior exposure to and training related to football equipment removal. We hypothesized that participation in the workshop would result in increased confidence and comfort in managing these scenarios.

## Materials and methods

Study design and setting

We conducted a pre-post quasi-experimental study at the University of Texas Southwestern, a three-year EM residency program in Dallas, Texas. All categorical residents were eligible to participate, and residents were recruited via email. In the absence of prior studies or preliminary data, a power analysis could not be performed to determine an appropriate sample size. These results will be used to inform power calculations for subsequent studies. The study was conducted on June 5, 2025. The institutional review board determined this study to be exempt.

Study protocol

An educational workshop on emergency football equipment removal was created to teach and practice hands-on skills. Participants first completed a pre-intervention survey to establish their baseline comfort and confidence levels and evaluate their exposure to football-related trauma injuries. The educational intervention consisted of a two-minute instructional video on emergency football equipment removal developed by the New Hampshire Musculoskeletal Institute and Safe Sports Network and was selected for its concise overview of emergency management from a reliable, nonprofit organization dedicated to safety in youth sports [8]. This was followed by a 30-minute, faculty-led hands-on workshop with two adult CPR manikins fitted with high school-level football helmets and shoulder pads. Two small groups of up to four residents were randomized to faculty facilitators who were EM physicians with fellowship training in sports medicine. Using a standardized teaching approach, faculty covered indications for equipment removal, facemask removal, cervical spine stabilization, helmet and shoulder pad removal, and post-removal assessment. Participants were provided multiple opportunities for hands-on practice and given real-time feedback throughout the session.

Outcome measures

Our primary outcome was measuring changes in residents’ self-reported confidence and comfort level in removing football equipment following the intervention. Secondary outcomes included assessing residents’ prior exposure and training related to football equipment removal during residency. Survey items were developed by the study team using recommendations from the National Athletic Trainers' Association (NATA) consensus guidelines and were reviewed for content relevance by faculty with sports medicine expertise. The survey instrument had not undergone formal validation, and reliability testing was not performed prior to study implementation. Self-reported measures of comfort and confidence with football equipment removal using a 5-point Likert scale (1= very uncomfortable/not confident; 5= very comfortable/very confident) were used. In addition, sociodemographic data was collected, and participants were assigned unique identifiers to match responses while maintaining anonymity.

In the absence of prior studies evaluating this educational intervention, no validated minimal meaningful change in confidence existed. Therefore, the study team selected a priori a 1.5-point increase on the 5-point Likert scale as a pragmatic benchmark representing a substantial improvement in perceived confidence. This threshold was used descriptively and was not intended as a validated measure of educational significance. 

Data collection and analysis

Data was collected and managed using REDCap electronic data capture tools (https://projectredcap.org/) hosted at The University of Texas Southwestern Medical Center [9]. Descriptive statistics were used to summarize resident responses. Paired ordinal data were analyzed using Wilcoxon signed-rank tests. Statistical significance was set at p-value < 0.05. Analyses were performed using Stata software (StataCorp LP, College Station, TX). All participants were included in the sociodemographic analysis, and only participants who completed both pre- and post-intervention surveys were included in paired analyses. Participants with incomplete or unmatched survey responses were excluded from that respective item analysis. 

## Results

Of 76 eligible EM residents, 29 (38%) participated, and 23 (79%) completed both the pre- and post-surveys. Participants included 17 postgraduate year (PGY)-1 (59%), 5 PGY-2 (17%), and 7 PGY-3 (24%). Despite most residents reporting being moderately to very familiar with the game of football (79%), many reported having little to no familiarity with current guidelines/best practices for safe equipment removal (69%). Only four residents (14%) reported ever being involved in a trauma cause that required equipment removal, 23 (82%) reported having never had formal training or instruction in equipment removal, and only five (18%) reported having had some prior simulation-based training (Table 1).

**Table 1:** Demographic, experience, and training characteristics of resident survey respondents PGY= postgraduate year. Values are number of respondents. Familiarity was assessed using a 5-point Likert scale. Training categories were not mutually exclusive.

Variable	PGY-1 (n =17)	PGY-2 (n = 5)	PGY-3 (n = 7)	Total (N = 29)
Involvement in trauma case requiring removal of football equipment				
0 cases	16	4	5	25
1-2 cases	1	1	2	4
3-5 cases	0	0	0	0
>5 cases	0	0	0	0
Familiarity with American Football				
Not familiar at all	1	0	0	1
Slightly familiar	2	0	1	3
Somewhat familiar	1	0	1	2
Moderately familiar	3	2	1	6
Very familiar	10	3	4	17
Familiarity with guideline and techniques for removing football equipment				
Not familiar at all	9	0	3	12
Slightly familiar	4	3	1	8
Somewhat familiar	3	2	3	8
Moderately familiar	1	0	0	1
Very familiar	0	0	0	0
Formal training or instruction in removing football equipment				
No formal training	14	3	6	23
Didactic lecture	0	0	0	0
Simulation-based training	3	1	1	5
Bedside teaching	0	0	0	0

**Figure 1:**
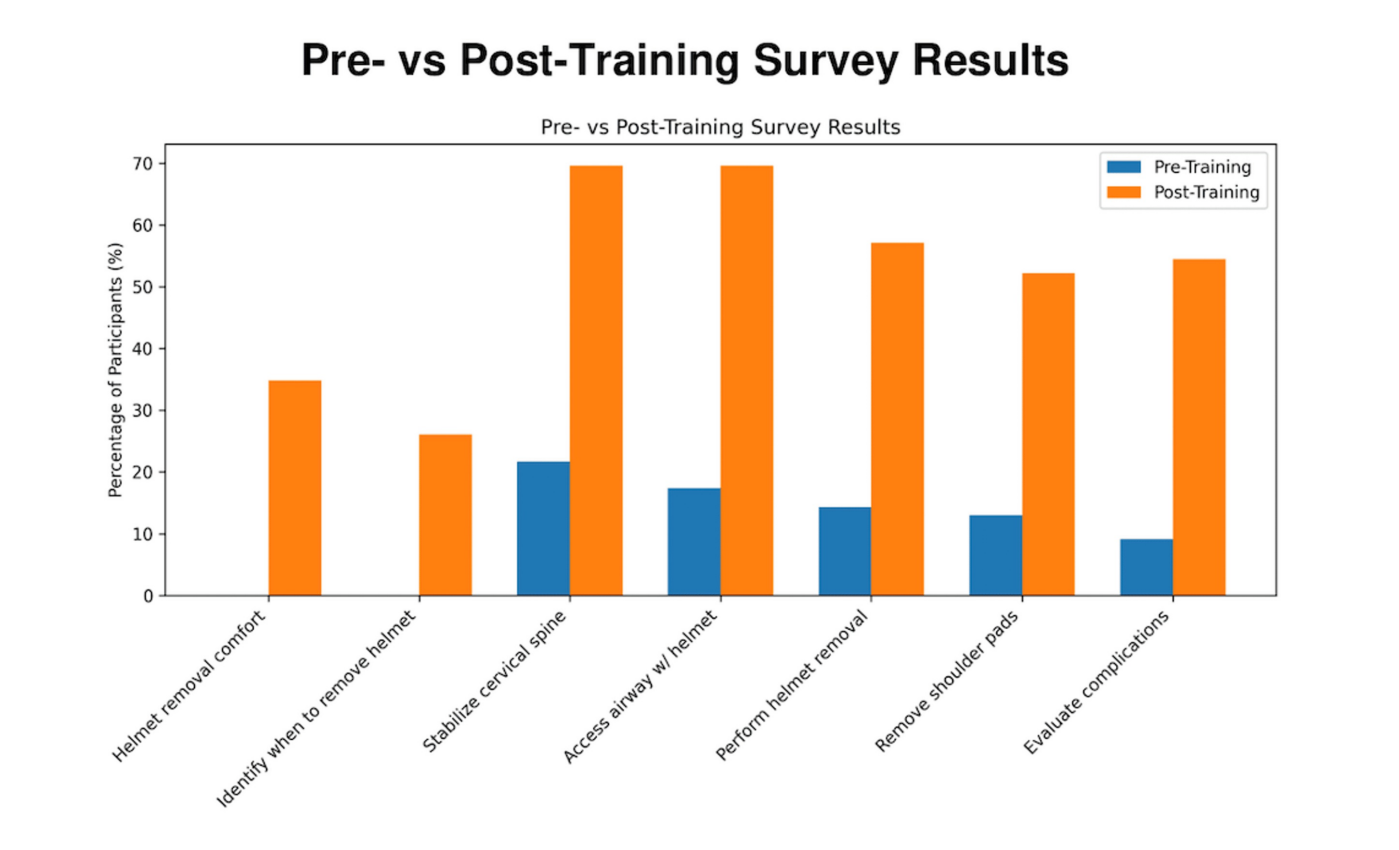
Participants reporting the highest level of comfort or confidence pre- and post-survey Percentage of participants reporting the highest level of comfort or confidence (“Very Confident or Very Comfortable”) for each survey item before and after the training intervention. Pre-training respondents (n = 23); post-training respondents (n = 23)

Following the intervention, residents reported a significant increase in levels of self-confidence across all assessed domains, including identifying the appropriate timing for equipment removal, stabilizing the cervical spine, accessing the airway, removing the helmet and shoulder pads, and evaluating for post-removal complications (p<.001). Self-reported comfort with removing football equipment in a trauma setting also increased significantly (p<.001; Figure 1, Table 2).

**Table 2:** Pre- and post-training confidence and comfort scores related to football equipment removal procedures (N = 23) Note: N = number of paired observations analyzed for each outcome. Scores are reported on a 5-point Likert scale (1 = not confident/comfortable at all; 5 = very confident/comfortable). Pre- and post-training comparisons were analyzed using the Wilcoxon signed-rank test. z = standardized Wilcoxon signed-rank test statistic. Variation in sample size reflects incomplete responses for individual survey items.

Outcome	N	Pre-training Mean (95% CI)	Post-training Mean (95% CI)	z	p value
Comfort with helmet/shoulder pad removal in a trauma	23	2.04 (1.71 – 2.38)	4.35 (4.14 - 4.56)	-4.25	< 0.001
Confidence identifying when helmet removal is indicated	23	1.87 (1.52 - 2.22)	4.13 (3.86 - 4.40)	-4.24	< 0.001
Confidence stabilizing cervical spine	23	3.39 (2.86 - 3.92)	4.65 (4.40 - 4.90)	-3.85	< 0.001
Confidence accessing airway with helmet in place	23	2.39 (1.93 - 2.86)	4.61 (4.32 - 4.89)	-4.23	< 0.001
Confidence performing helmet removal while maintaining spinal alignment	21	1.86 (1.44 - 2.27)	4.52 (4.25 - 4.80)	-4.05	< 0.001
Confidence performing shoulder pad removal while in spinal alignment	23	1.87 (1.52 - 2.22)	4.48 (4.22 - 4.73)	-4.25	< 0.001
Confidence evaluating post-removal complications	22	2.23 (1.80 - 2.66)	4.55 (4.32 - 4.77)	-4.12	< 0.001

## Discussion

This pilot study demonstrates that a brief, simulation-based educational intervention significantly improves EM residents’ self-reported confidence and comfort in football equipment removal. These findings align with prior literature supporting simulation as an effective educational tool for addressing gaps in resident exposure to uncommon but high-stakes clinical scenarios and in practicing high-risk, low-frequency procedures in a low-stakes environment [10-12]. Although trauma and spine management are required competencies that all EM residencies must incorporate into their curriculum per The Model of the Clinical Practice of Emergency Medicine, exposure to removing football equipment or managing athletes with suspected SCI can vary [13-15]. Our findings highlight variability in resident experience even within a single program. Incorporating structured workshops into residency curricula may provide a practical approach to standardizing exposure and improving preparedness for these high-acuity situations.

Limitations

This study has several limitations. In the absence of prior studies or preliminary data on this subject, a power analysis could not be performed to determine an appropriate sample size. Our small sample size and single-center design may limit the generalizability of these findings. Since participation was voluntary, residents already interested in sports medicine may have been more likely to participate, potentially introducing selection bias. Additionally, the predominance of PGY-1 participants could have impacted survey results, as many interns do not have as much clinical experience as compared to their PGY-2 and PGY-3 peers. Depending on the level of training or time of year, different rotations, including trauma surgery, could contribute to equipment removal skills. Future work broadening participant populations to increase representation from senior-level residents, enrolling pediatric EM fellows, or involving orthopedic trainees could add to future studies. 

Our survey had many limitations as well. First, it was not formally validated or assessed for reliability. Next, it was also administered immediately post-intervention, introducing potential recency bias and therefore long-term retention of confidence in performing this skill is unknown. Most importantly, it did not measure objective performance data of the skills performed, but rather subjective self-reported outcomes. Improvements in self-reported confidence do not necessarily translate into procedural competency or improved patient outcomes; future studies should evaluate objective performance measures, such as degree of cervical spine motion, inclusion of a control group, or knowledge retention [16]. Lastly, the use of a manikin instead of a human model may not fully replicate real-world conditions, and follow-up studies should instead utilize human models.

## Conclusions

Emergency medicine residents report limited exposure to the removal of football equipment in athletes with suspected SCI. Although these ED presentations are rare, appropriate knowledge and readiness when managing these athletes can improve the quality of care and outcomes in these patients. A brief, hands-on educational workshop was associated with improved self-reported confidence and comfort in managing these patients. This pilot intervention may help address gaps in training for rare but critical scenarios and serve as a foundation for future research evaluating skill performance and long-term educational outcomes.

## Appendices

Section A

Pre-survey: Comfort Level with Football Pad Removal in Trauma

The purpose of this survey is to evaluate your comfort and experience with the procedure for removing football equipment in the context of trauma. Your responses will help identify areas for further training and improve procedural confidence.

Number Identifier: ______

            1.          Level of Training:

            •           PGY-1

            •           PGY-2

            •           PGY-3

            2.          How many times have you been involved in a trauma case requiring the removal of  football equipment?

            •           0

            •           1-2

            •           3-5

            •           More than 5

            3.          How familiar are you with the game of American football?

            •           Not familiar at all

            •           Slightly familiar

            •           Somewhat familiar

            •           Moderately familiar

            •           Very familiar

            4.          How familiar are you with the guidelines and techniques for removing football equipment in trauma?

            •           Not familiar at all

            •           Slightly familiar

            •           Somewhat familiar

            •           Moderately familiar

            •           Very familiar

            5.          Have you received any formal training or instruction on football equipment removal in trauma? (Select all that apply)

            •           No formal training

            •           Didactic lecture

            •           Simulation-based training

            •           Bedside teaching 

            •           Other (please specify)

            6.          Rate your comfort level with the procedure for removing a football helmet and shoulder pads in trauma:

            •           Very uncomfortable

            •           Uncomfortable

            •           Neutral

            •           Comfortable

            •           Very comfortable

            7.          How confident are you in identifying when it is appropriate to remove the helmet versus leaving it in place?

            •           Not confident at all

            •           Slightly confident

            •           Somewhat confident

            •           Moderately confident

            •           Very confident

            8.          Rate your confidence in performing the following steps during football equipment removal in a trauma scenario:

            •           Stabilizing the cervical spine:

            •           Not confident, Slightly confident, Somewhat confident, Moderately confident, Very confident

            •           Accessing the airway while the helmet is in place:

            •           Not confident, Slightly confident, Somewhat confident, Moderately confident, Very confident

            •           Performing the actual removal of the helmet while maintaining spinal alignment:

            •           Not confident, Slightly confident, Somewhat confident, Moderately confident, Very confident

            •           Performing the removal of the shoulder pads while in spinal alignment:

            •           Not confident, Slightly confident, Somewhat confident, Moderately confident, Very confident

            •           Evaluating the patient for potential complications after helmet removal:

            •           Not confident, Slightly confident, Somewhat confident, Moderately confident, Very confident

Section B

Post- Survey: Comfort Level with Football Pad Removal in Trauma

The purpose of this survey is to evaluate your comfort and experience with the procedure for removing football equipment in the context of trauma. Your responses will help identify areas for further training and improve procedural confidence.

Number Identifier: _____

            1.          Level of Training:

            •           PGY-1

            •           PGY-2

            •           PGY-3

            2.          Rate your comfort level with the procedure for removing a football helmet and shoulder pads in trauma:

            •           Very uncomfortable

            •           Uncomfortable

            •           Neutral

            •           Comfortable

            •           Very comfortable

            3.          How confident are you in identifying when it is appropriate to remove the helmet versus leaving it in place?

            •           Not confident at all

            •           Slightly confident

            •           Somewhat confident

            •           Moderately confident

            •           Very confident

            4.          Rate your confidence in performing the following steps during football equipment removal in a trauma scenario:

            •           Stabilizing the cervical spine:

            •           Not confident, Slightly confident, Somewhat confident, Moderately confident, Very confident

            •           Accessing the airway while the helmet is in place:

            •           Not confident, Slightly confident, Somewhat confident, Moderately confident, Very confident

            •           Performing the actual removal of the helmet while maintaining spinal alignment:

            •           Not confident, Slightly confident, Somewhat confident, Moderately confident, Very confident

            •          Performing the removal of the shoulder pads while in spinal alignment:

            •           Not confident, Slightly confident, Somewhat confident, Moderately confident, Very confident

            •           Evaluating the patient for potential complications after helmet removal:

            •           Not confident, Slightly confident, Somewhat confident, Moderately confident, Very confident
